# Opportunistic Genetic Screening for Familial Hypercholesterolemia in Heart Transplant Patients

**DOI:** 10.3390/jcm12031233

**Published:** 2023-02-03

**Authors:** María Salgado, Beatriz Díaz-Molina, Elías Cuesta-Llavona, Andrea Aparicio, María Fernández, Vanesa Alonso, Pablo Avanzas, Isaac Pascual, David Neuhalfen, Eliecer Coto, Juan Gómez, Rebeca Lorca

**Affiliations:** 1 Área del Corazón, Hospital Universitario Central Asturias, 33011 Oviedo, Spain; 2 Unidad de Insuficiencia Cardiaca Avanzada y Trasplante, Área del Corazón, Hospital Universitario Central Asturias, 33011 Oviedo, Spain; 3 Instituto de Investigación Sanitaria del Principado de Asturias, ISPA, 33011 Oviedo, Spain; 4 Departamento de Medicina, Universidad de Oviedo, 33003 Oviedo, Spain; 5 Unidad de Cardiopatías Familiares, Área del Corazón y Departamento de Genética Molecular, Hospital Universitario Central Asturias, 33011 Oviedo, Spain; 6 Redes de Investigación Cooperativa Orientadas a Resultados en Salud (RICORs), 28029 Madrid, Spain; 7 CIBER-Enfermedades Respiratorias, 28029 Madrid, Spain; 8 Departamento de Morfología y Biología Celular, Universidad de Oviedo, 33003 Oviedo, Spain

**Keywords:** familial hypercholesterolemia (FH), genetic testing, inherited cardiac conditions, heart failure (HF)

## Abstract

Heart transplantation remains the gold standard for the treatment of advanced heart failure (HF). Identification of the etiology of HF is mandatory, as the specific pathology can determine subsequent treatment. Early identification of familial hypercholesterolemia (FH), the most common genetic disorder associated with premature cardiovascular disease, has a potential important impact on clinical management and public health. We evaluated the genetic information in the genes associated with FH in a cohort of 140 heart-transplanted patients. All patients underwent NGS genetic testing including *LDLR, APOB*, and *PCSK9*. We identified four carriers of rare pathogenic variants in *LDLR* and *APOB*. Although all four identified carriers had dyslipidemia, only the one carrying the pathogenic variant *LDLR* c.676T>C was transplanted due to CAD. Another patient with heart valvular disease was carrier of the controversial *LDLR* c.2096C>T. Two additional patients with non-ischemic dilated cardiomyopathy were carriers of variants in *APOB* (c.4672A>G and c.5600G>A). In our cohort, we identified the genetic cause of FH in patients that otherwise would not have been diagnosed. Opportunistic genetic testing for FH provides important information to perform personalized medicine and risk stratification not only for patients but also for relatives at concealed high cardiovascular risk. Including the *LDLR* gene in standard NGS cardiovascular diagnostics panels should be considered.

## 1. Introduction

Heart transplantation remains the gold standard treatment of advanced heart failure (HF), in the absence of contraindications [1]. The risk of developing HF is multifactorial, with inherited and acquired risk factors. Recent European guidelines for the diagnosis and treatment of acute and chronic HF [1] reinforce the message that identification of the etiology is mandatory, as the specific pathology can determine subsequent treatment.

In this sense, investigating the genetic causes of possible inherited cardiomyopathies is of utmost importance. For instance, a genetic cause can be identified in up to 40% of patients with dilated cardiomyopathy (MCD) [1]. Finding a pathogenic gene variant in a patient with inherited cardiomyopathies allows for better prediction of the disease outcome and progression, may contribute to indications for device implantation and inform genetic counseling for families [1]. Moreover, early identification of asymptomatic relatives may lead to early treatment and prevention of progression to HF and proper genetic counseling [1]. In this regard, our group already performed genetic testing on a cohort of transplanted patients and successfully identified concealed inherited cardiomyopathies such as MCD and hypertrophic cardiomyopathy (MCH) [2]. We found a pathogenic variant in genes associated with MCH and MCD (*FLNC, LMNA, MYBPC3, MYH7, TNNT2*, and *TTN)* in nearly 1 out of every 10 transplanted recipients [2].

However, nowadays, in Western and developed countries, coronary artery disease (CAD) is one of the predominant causes of HF [3]. Atherosclerotic cardiovascular (CV) disease (ASCVD) is known to be a major cause of morbidity and mortality [4]. As a result, the latest European guidelines on CV disease prevention highlight the importance of a healthy lifestyle without smoking and controlling all CV risk factors [4]. In this regard, dyslipidemia is one of the main causal and modifiable CV risk factors [4] and may also be the result of an inherited genetic disorder: familial hypercholesterolemia (FH).

Early identification of FH has a potential important impact on clinical management and public health [5]. Young adults with FH, if untreated, could experience a 90-fold increase in ASCVD mortality [6]. However, FH is still severely underdiagnosed [7]. FH can be diagnosed based on clinical findings alone. Nonetheless, genetic information can be key to achieving a definite diagnosis [8] and could also be considered the “gold standard” for FH diagnosis [9].

In this regard, we believe that opportunistic genetic screening for FH could be extremely useful. Identification of a pathogenic variant in a patient with FH ensures FH diagnosis and enables cascade family genetic screening. This may help in early identification of asymptomatic relatives in which early treatment and prevention strategies, as well as proper genetic counseling, could subsequently be performed [1]. Therefore, in this study, we aimed to evaluate the genetic information in the genes associated with FH in a cohort of heart-transplanted patients who had already undergone genetic testing to identify other underlying inherited cardiomyopathies [2].

## 2. Materials and Methods

### 2.1. Patients

This is a retrospective study of 140 heart-transplanted patients who agreed to undergo genetic study. All participants were transplanted in the same reference center for cardiac transplant from 2003 to 2018, Hospital Universitario Central Asturias (HUCA). Most transplanted patients were male (74%), and the mean age of the cohort was 55 years (±9.7 SD) [2]. Patients were of European ancestry and from the region of Asturias, northern Spain (total population, approx. 1 million).

All recruited participants signed informed consent for genetic testing. The institutional Ethical Committee evaluated and approved this study (CEImPA 2022.254).

### 2.2. Genetic Study and Variant Classification

DNA was obtained from blood leukocytes by the salting-out method in all patients, and NGS was performed with a 205 cardiovascular-disease-associated gene panel, as previously reported [2]. DNA was sequenced by ion torrent technology using semiconductor chips in an Ion Gene Studio S5 Plus sequencer (Termo Fisher Scientific) according to a previously described procedure [10,11,12,13]. Raw data were processed with the Torrent Suite v5 pipeline. Variant Caller v5 software was used for variant calling. For variant annotation, including population, functional, disease-related, and in silico predictive algorithms, we used Ion Reporter (Thermo Fisher Scientific) and HD Genome One (DREAMgenics S.L.) software. To analyze sequence quality and depth coverage and for variant identification, we used the Integrative Genome Viewer (IGV, Broad Institute) [2].

In this study, we evaluated all variants in genes associated with familial hypercholesterolemia: *LDLR, APOB, PCSK9* (heterozygous FH), and *LDLRAP1* (autosomal recessive FH). Only genetic variants with an allele frequency < 0.01 were evaluated. Clinical variant interpretation was performed according to the American College of Medical Genetics and Genomics (ACMG-AMP) criteria [14]. Only variants classified as pathogenic/likely pathogenic (P/LP) or variants of uncertain significance (VUS) were reported. Variants classified as benign or likely benign were ignored. Sanger sequencing was performed to confirm all reported variants. Family screening was offered if P/LP variants were identified.

### 2.3. Clinical Information

We reviewed all available clinical records form patients carrying P/LP or VUS variants in the genes related to FH. We reviewed their birth data, clinical evolution, and all cardiovascular risk factors (CVRFs): high blood pressure or hypertension (HTN), tobacco consumption, diabetes mellitus (DM), and dyslipidemia (DL). Based on their clinical records, the Dutch Lipid Clinical Network (DLCN) score was evaluated [4]. Low-density lipoprotein cholesterol (LDLc) levels and medical treatment, including lipid-lowering therapy and immunosuppressive drugs, were also reviewed. Personal and familial ASCVD data were also collected, considering “premature ASCVD” cutting age according to the DLCN definition [4]. Other vascular beds of ASCVD beyond CAD were considered.

## 3. Results

Clinical characteristics of the transplanted cohort (140 patients) were explained elsewhere [2]. Nearly half of patients (45%) were transplanted due to CAD. Most patients transplanted due to ischemic cardiomyopathy were previous smokers (81%) and/or suffered from HTN (62%). Moreover, nearly one of every two patients had been diagnosed with dyslipidemia (48%) before cardiac transplantation. In contrast, the rate of personal history of diabetes mellitus was low.

Genetic testing of this cohort revealed four rare genetic variants in two genes related to familial hypercholesterolemia (Table 1).

All four patients were male (Table 2). Although all of them had a prior history of dyslipidemia, atherosclerosis with demonstrated coronary artery disease by coronary angiogram was the etiology of the heart dysfunction in only one (Patient 1).

Patient 1 was transplanted in our institution in 2001 due to ischemic cardiac disease. He had all major CVFRs: tobacco consumption, HTN, DM, and DL. He had a personal history of a premature CV disease, as he presented with a myocardial infarction before the age of 55, when he was 43 years old. He also suffered from atherosclerosis at other vascular levels, such as carotids. Considering his highest recorded LDLc levels, DLCN score was, at least, possible. Genetic testing revealed that he was a carrier of a the pathogenic variant *LDLR* c.676T>C. This is pathogenic variant, which was absent in the control cohorts, was previously reported in the literature [15,16,17,18]. The last recorded LDLc levels of Patient 1, despite being on statin treatment, were above the objective threshold (Table 2). Moreover, he presented with severe cardiac allograft vasculopathy during follow-up. Patient 1 moved from our region, so no further cardiological follow-ups were available to review. Unfortunately, he died in 2013, 12 years after heart transplant.

**Figure 1:**
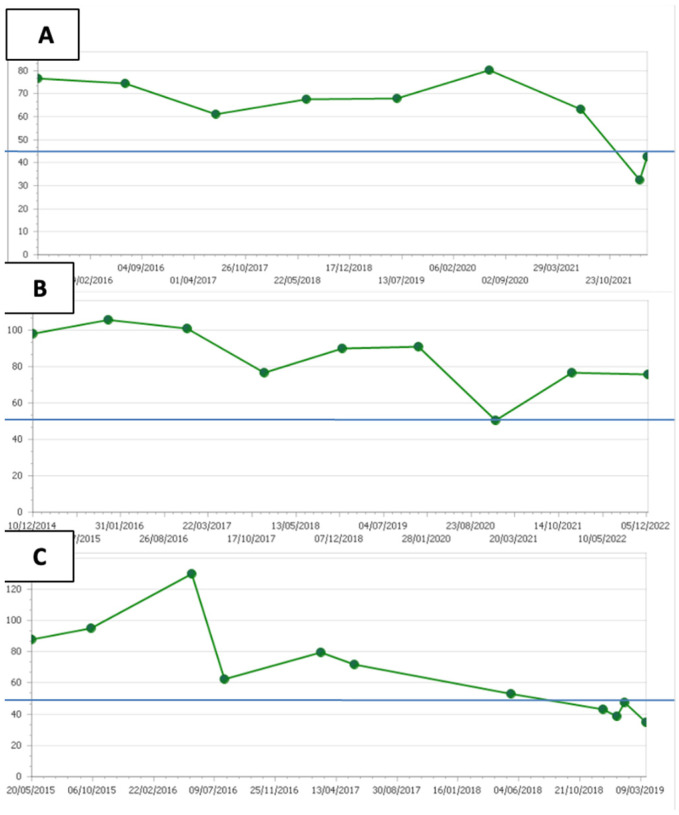
LDLc levels from 2015 to date. (**A**) Patient 2; (**B**) Patient 3; (**C**) Patient 4.

Patient 2 was a male individual with a long history of valvular cardiac disease due to rheumatic fever that required heart valve surgery in 1997. However, he developed severe symptomatic ventricular dysfunction. As a result, he received a cardiac transplant in 2011. He had a prior history of dyslipidemia and was on statin treatment since 1995. His sister also had a history of dyslipidemia. Although he had an LDLc of 114 mg/dL in 2013, better control of LDLc levels were achieved afterwards (Figure 1A), falling below 55 mg/dL at the time of the last follow-up in 2022 (Table 1). No significant atherosclerosis disease was reported in this patient. He died in 2022 due a respiratory failure secondary to COVID19 infection. Patient 2 was carrier of a rare variant in the *LDLR* gene (*LDLR* c.2096C>T, p.P699L; also reported as p.Pro678Leu in the literature). The substitution from a C to T in this position results in a change from proline to leucine. This takes place in the third EGF-like repeat within the LDLreceptor EGF precursor homology domain. Most in silico scores predict that this missense change has a damaging effect in the synthetized protein. *LDLR* p.P699L has been identified at a very low allele frequency (0.00003895) in GnomAD (http://gnomad.broadinstitute.org). Given the high prevalence of FH, we believe that this low frequency could be considered consistent with the disease. Moreover, *LDLR* c.2096C>T hasbeen previously reported in FH patients [16,19,20,21,22,23,24,25], and many submissions to ClinVar have classified it as either pathogenic or likely pathogenic. However, a laboratory of genetics and molecular cardiology reported a lack of segregation in some families (https://erepo.clinicalgenome.org/evrepo/ui/interpretation/ea33bc95-d102-418a-b019-83d258dcad77). In summary, the global evidence is not sufficient to classify this variant as undoubtedly LP. Thus, it was considered VUS.

Patient 3 had a prior history of dyslipidemia and was under lipid-lowering treatment since the age of 30. Moreover, he had a family history of ischemic cardiomyopathy and hypercholesterolemia. However, he developed severe heart disease of non-ischemic origin. He underwent heart transplant in 2000 without CAD in this angiogram. Atherosclerosis was not reported in any other vascular territory. His last LDLc levels were variable during follow-up, with some values over 100 mg/dL but at least one around 55 mg/dL (Figure 1B). He was carrier of a missense variant in the *APOB* gene (c.4672A>G, p.T1558A). As a result, there was a change in the resulting APOB protein, with an alanine instead of a threonine at the 1558 position. This is a rare variant that is control cohort populations and has been reported as VUS in the ClinVar database. In silico scores are inconclusive, as they do not agree on the predictions regarding the possible impact onthe synthetized protein. In summary, due to insufficient evidence supporting the pathogenicity ofthis variant, its clinical significance remains uncertain, and it was classified as VUS.

Patient 4 suffered heart failure due to non-ischemic cardiomyopathy. He had a known history of dyslipidemia. Thanks tohigh-intensity lipid-lowering treatment, his last LDLc control levels were optimal (Figure 1C). CAD and atherosclerosis were not reported at other vascular locations. However, he developed enolic cirrhosis. Genetic testing revealed that he was a carrier of a rare APOB variant. *APOB* p.R1867Q (also known as c.5600G>A) was identified at a low frequency in control cohort populations (gnomAD, exomes 0.00004). Due to this missense change, at codon 1867, glutamine replaces an arginine. An alternate amino acid substitution at this position, *APOB* p.R1867W (c.5599C>T), was reported in an affected patient from a FH Iberian cohort [26]. In the ClinVar database, there are six submissions classifying *APOB* p.R1867Q as VUS. Predictions based on computational tools do not support its pathogenicity or show inconclusive results. As a result, due to insufficient evidence at this time, the variant was considered VUS.

## 4. Discussion

FH is one of the most common genetic conditions and the main genetic disorder associated with premature ASCVD [5,12,27,28,29], with a prevalence of 1/250–1/500 individuals [5,28,29]. However, FH is not only clinically overlooked [7] but also genetically underdiagnosed.Current European guidelines consider FH patients directly as CV high-risk patients [4]. In fact, if FH patients remain untreated, they could suffer a CV event and even death by the second decade of life [30]. Nonetheless, if FH patients are identified in their early life, initiation of lipid-lowering treatment can substantially reduce their risk of ASCVD [31]. In this regard, in our cohort, Patient 3 (carrier of *APOB* c.4672A>G), who had been on statin treatment since the age of 30 and subsequently achieved target LDLc levels, presented no signs of atherosclerotic disease on his pretransplant coronary angiogram.

Genetic screening for FH should include at least the three primary genes associated with heterozygous autosomal dominant FH: *LDLR, APOB*, and *PCSK9* [8]. Most reported FH pathogenic variants are identified in the *LDLR* gene (>90%) and can be produced by numerous mechanisms including both nonsense and missense variants [32,33]. In our transplant cohort, we identified a patient harboring the known pathogenic variant *LDLR* c.676T>C. This variant was previously reported in our country in association with FH [18]. Patient 1 had a known history of dyslipidemiaas important CVRF, which was to blame for his CAD. Furthermore, thanks to statin treatment, his LDLc levels were not surprisingly high. Thus, had genetic screening for HF not been performed, his diagnosis would have been missed, and family screening could not have been advised.

Moreover, the other *LDLR* variant was identified in Patient 2, a male individual with valvular heart disease due to rheumatic fever. Therefore, although he had a prior history of dyslipidemia and was under lipid-lowering treatment since 1995, he had no personal history of ASCVD, and clinical focus was not mainly on LDLc levels. However, thanks to lipid-lowering treatment, globally, he achieved good LDLc control levels (Figure 1A). In contrast to Patient 1, genetic information of Patient 2 should be evaluated with caution. The *LDLR* c.2096C>T variant, has been previously reported in many studies in association with FH [16,19,20,21,22,23,24,25]. However, the definite clinical significance of this remains unclear.

In this sense, interpreting the clinical significance of rare variants in the *APOB* gene is even more difficult that in those reported in the *LDLR* gene. The most frequent *APOB* pathogenic variant in Europe is *APOB* p.Arg3527Gln [34]. However, establishing causality in other *APOB* variants remains particularly challenging [35,36,37,38,39]. In this study, we identified two variants affecting different regions of *APOB* that were not present in healthy control populations nor reported in the literature. Patient 3, a carrier of *APOB* c.4672A>G, had a suggestive personal and family history of FH. On the other hand, the last patient, a carrier of *APOB* c.5600G>A, had dyslipidemia without any other known personal or family history suggestive of FH. In both cases, pending additional studies, APOB variants were classified as VUS.

To date, only a few studies have provided genetic information about the genetic status of heart transplant recipients. They all provided interesting information about inherited cardiomyopathies, with a high genetic yield that improves depending on the cohort selection and the genes included in their NGS genetic panels [2,40,41,42,43,44,45]. However, all such studies focused on inherited cardiomyopathies behind non-ischemic DCM, and none provided additional genetic information. To the best of our knowledge, this is the first study reporting genetic opportunistic screening of FH in transplanted recipients. The genetic yield for FH was also high. Given the already high prevalence of FH in the general population and that 45% of this cohort had been transplanted due to CAD, the high percentage is not surprising. As a result, we believe that implementing the *LDLR* gene in the targeted gene panel for NGS sequencing in this group of patients could be worthwhile.

Genetic testing results provide prognostic information to develop personalized medicine and personalized risk stratification [8]. Moreover, if a pathogenic variant is identified in the index case, epidemiological and cost analysisdata support familial genetic screening for the disease [8]. Although heart transplantation remains the gold standard for the treatment of advanced heart failure [1], due the limited availability of donor organs and the associated global medical challenge, it is generally seen a resource of last resort. In this scenario, identification of genetic predisposition of actionable diseases such as FH is of utmost importance. Because the risk of myocardial infarction in properly treated patients with FH could be equal to that of the general population [31], the rate of heart transplantations in patients with FH should also not differ from that of the general population. As a result, early identification and treatment of FH patients could increase the availability of heart transplantation for other indications that are not as easily preventable.

On the other hand, heart transplant recipients suffer from cardiac allograft vasculopathy (CAV) [46], a unique form of accelerated atherosclerosis [47]. Interestingly, Patient 1 suffered severe CAV. Although the etiology of CAV etiology can be multifactorial, traditional CVRF risk factors for CAD, specially dyslipidemia, are thought to contribute [48,49,50,51]. On this basis, statins are already recommended for heart transplant recipients in routine treatment [52]. Unfortunately, side effects and interactions with immunosuppressants sometimes limit their optimal use in such patients [53]. Therefore, it may be informative to include in the genetic study of FH in pretransplant screening in order to identify individuals at higher risk, such as Patient 1. The availability of proprotein convertase subtilisin/kexin type-9 inhibitors (PCSK9i) may be of use. Some studies have already provided data supporting their efficacy and short-term safety in cardiac transplant recipients [54,55]. As a result, although more information about PCSK9i safety in heart transplant patients is needed, those with FH may benefit from early initiation of this therapy.

## 5. Limitations

The classification of genetic variants may change over time depending on emerging data and newly available resources. Gene dosage was not routinely preformed in this cohort of patients for the identification of large deletions. LDLc levels and/or DLCN scores from the whole cohort were not evaluated. As a result, the pretest probability of the transplanted cohort is unknown.

## 6. Conclusions

In this cohort of heart transplant patients, we identified the genetic cause of familial hypercholesterolemia in dyslipidemia patients that otherwise would not have been diagnosed. Opportunistic genetic testing for familial hypercholesterolemia provides important information to perform personalized medicine and risk stratification. Including the *LDLR* gene in standard NGS cardiovascular diagnostics panels should be considered. Results from genetic testing enable cascade family testing. Therefore, prevention strategies can be extended to all available relatives at concealed high cardiovascular risk.

## Figures and Tables

**Table 1:** Rare genetic variants found in the cohort by *LDLR*, *APOB*, and *PCSK9* gene sequencing.

Number	Gene	cDNA	Exon	Protein	RefSeq Transcript	Effect
1	*LDLR*	c.676T>C	4	p.S226P	NM_000527	Missense
2	*LDLR*	c.2096C>T	14	p.P699L	NM_000527	Missense
3	*APOB*	c.4672A>G	26	p.T1558A	NM_000384	Missense
4	*APOB*	c.5600G>A	26	p.R1867Q	NM_000384	Missense

**Table 2:** Clinical characteristics of patients harboring rare variants associated with familial hypercholesterolemia.

Patient	Genetic Variant	Sex	Birth Date	Heart Transplant	Cardiopathy	DL	Provided Lipid-Lowering Treatment	Last LDL (mg/dL)
1	*LDLR* c.676T>C	Male	1956	2001	Ischemic	Yes	Statin	156
2	*LDLR* c.2096C>T	Male	1942	2011	Valvular	Yes	Statin	<55
3	*APOB* c.4672A>G	Male	1969	2000	Non-ischemic DCM	Yes	Statin	78
4	*APOB* c.5600G>A	Male	1977	2007	Non-ischemic DCM	Yes	Statin	<55

DCM: dilated cardiomyopathy; DL: dyslipidemia.
